# Patient knowledge of fecal calprotectin in inflammatory bowel disease (IBD): An observational study in Mexico

**DOI:** 10.12688/f1000research.27629.2

**Published:** 2021-02-05

**Authors:** Brenda Maldonado-Arriaga, Sergio Sandoval-Jiménez, Juan Rodríguez-Silverio, Sofía Lizeth Alcaráz- Estrada, Tomás Cortés-Espinosa, Rebeca Pérez-Cabeza de Vaca, Jonathan Shaw, Paul Mondragón-Terán, Cecilia Hernández-Cortez, Juan Antonio Suárez-Cuenca, Graciela Castro-Escarpulli

**Affiliations:** 1 Laboratorio de Metabolismo Experimental e Investigación Clínica; División de Investigación Clínica, ISSSTE, Félix Cuevas 540, Col del Valle Sur, Benito Juárez, Ciudad de México, 03229, Mexico; 2 Hospital General de 2A Troncoso, Instituto Mexicano del Seguro Social, Ciudad de México, Mexico; 3 Laboratorio de Investigación Clínica y Ambiental. Departamento de Microbiología, Escuela Nacional de Ciencias Biológicas, Instituto Politécnico Nacional, Calle Plan de Ayala s/n, Santo Tomás, Miguel Hidalgo, Ciudad de México, 11340, Mexico; 4 Escuela Superior de Medicina, Instituto Politécnico Nacional, Miguel Hidalgo, Ciudad de México, 11340, Mexico; 5 Unidad de Análisis y Referencia Virológica, ISSSTE, Félix Cuevas 540, Col del Valle Sur, Benito Juárez, Ciudad de México, 03229, Mexico; 6 Clínica de Enfermedad Inflamatoria Intestinal, Servicio de Gastroenterología, ISSSTE, Félix Cuevas 540, Col del Valle Sur, Benito Juárez, Ciudad de México, 03229, Mexico; 7 Coordinación de Investigación y División de Investigación Biomédica, ISSSTE, Félix Cuevas 540, Col del Valle Sur, Benito Juárez, Ciudad de México, 03229, Mexico; 8 Department of Infection, Immunity & Cardiovascular Disease, University of Sheffield Medical School, Broomhall, Sheffield, S10 2TG, UK; 9 Laboratorio de Bioquímica Microbiana, Departamento de Microbiología, Escuela Nacional de Ciencias Biológicas, Instituto Politécnico Nacional, Calle Plan de Ayala s/n, Santo Tomás, Miguel Hidalgo, Ciudad de México, 11340, Mexico

**Keywords:** Inflammatory bowel disease; Fecal calprotectin; Level of knowledge; Ulcerative colitis; Crohn’s disease.

## Abstract

**Background:** Fecal calprotectin (FC) can be a valuable tool to optimize health care for patients with inflammatory bowel disease (IBD). The objective of this observational study was to determine the level of knowledge of the FC test in Mexican patients with IBD.

**Methods:** A self-report questionnaire was distributed via Facebook to patients with IBD. The survey consisted of 15 questions in two categories: the first category assessed knowledge of IBD diagnosis, and the second category assessed knowledge of the FC test.

**Results:** In total, 460 patients with IBD participated, of which 83.9% (386) had ulcerative colitis (UC) and 16.0% (74) had Crohn’s disease (CD). Regarding IBD diagnosis, 41.9% of participants stated that they did not know of a non-invasive test for fecal matter to identify inflammation of the colon. Regarding the FC test, 57.5% (UC) and 58.1% (CD) stated that they did not know about the test. Additionally, 65.8% (UC) and 51.3% (CD) of participants stated that they had never received the FC test and 82.6% (UC) and 77.0% (CD) recognized that the FC test was difficult to access in their medical practice. Furthermore, 66% (UC) and 52.7% (CD) of participants noted that their specialist doctor had never suggested the FC test to them, yet 89.1% (UC) and 87.8% (CD) stated that they would prefer FC analysis for their IBD follow-up assessments.

**Conclusions:** There is little knowledge of the FC biomarker among Mexican patients with IBD. This suggests the need for greater dissemination of its use and scope as a biomarker in IBD.

## Abbreviations

IBD, Inflammatory bowel diseases; FC, Fecal Calprotectin; UC, Ulcerative Colitis; CD, Crohn’s Disease.

## Introduction

Inflammatory bowel disease (IBD) is defined as a group of chronic inflammatory disorders of unknown cause that affect the gastrointestinal tract and includes two diseases: Crohn’s disease (CD) and Ulcerative Colitis (UC). These diseases are defined according to clinical, radiological, endoscopic, and histological criteria, and are characterized by chronic relapses, which present in outbreaks (active phases) and periods of remission (inactive phases) ^1^.

Currently, gastrointestinal endoscopy, histological examination of biopsies, and contrast imaging are mandatory techniques for the diagnosis and evaluation of IBD activity. Due to the complexity of the disease and the need for a multidisciplinary approach, diagnosing and treating the condition is a challenge for medical specialists. Patients generally take five to ten years to be diagnosed, which implies that treatments are applied late and therefore, the quality of life of patients with IBD is directly affected ^2^. Patients with IBD also worry about a timely diagnosis, as well as complications of the disease and secondary conditions ^3, 4^.

Unfortunately, endoscopy is not easily accessible in many rural areas of the Mexican Republic and when performed are sometimes unnecessary, a problem compounded by the cost of the procedure. Recently, in different international institutions the use of biomarkers or biological markers has been routinely applied. The most prominent biomarker used for IBD is fecal calprotectin (FC), which is a protein derived from neutrophils, released in the feces in response to inflammation of the intestinal mucosa. FC levels have been found to be associated with endoscopic severity, prediction of mucosal healing, and prediction of relapse; therefore, it is a useful biomarker for monitoring patient response to treatment ^5, 6^. Additionally, a primary objective of the treatment of IBD is to improve the patient’s health-related quality of life regardless of the type of therapy used. Patient knowledge of alternative methods for diagnosis and follow-up of IBD leads to increased well-being of the patients and to a reduction in overall healthcare costs ^7^.

Despite the clinical utility of FC in IBD, the lack of dissemination and knowledge among patients could limit its acceptance and use as a clinical test. Currently in Mexico, there are no official statistics on the use of FC as a diagnostic marker and follow-up test for IBD. Consequently, the present study aims to assess knowledge regarding the use of FC as a diagnostic test among patients with IBD.

## Methods

### Study design

This study used an observational cross-sectional design. Through the “Fundación Vivir con Crohn y CUCI A.C” (a non-profit foundation that offers information and support to people with IBD in Mexico), an electronic questionnaire was disseminated with questions aimed at assessing participant knowledge of FC and its use in IBD treatment. The questionnaire was developed by “Fundación Vivir con Crohn y CUCI A.C”, gastroenterologists and health advocates of Hospital C.M.N. “20 de Noviembre”, ISSSTE, in 2020. Two gastroenterologists in the group validated the 15 questions. A pilot test of the questionnaire was carried out with 5% of the population (23 people with IBD who attended the hospital), to ensure the validity of the questionnaire. The questionnaire was anonymous and voluntary, and consisted of 15 basic questions about the patient’s social and demographic data, as well as questions related to knowledge of the FC test ^8^. A link to the questionnaire was posted on the “Fundación Vivir con Crohn y CUCI A.C”’s Facebook site on the 3 ^rd^ July 2020. The survey was hosted on Google Forms. 

### Participants

Inclusion criteria were Mexican adults, aged 18 to 45 years old and diagnosed with inflammatory bowel disease. The sample size was calculated using the central limit theorem ^9^ with a margin of error of 5% and a confidence interval of 95%. According to a previous study by Yamamoto *et al.*, 2015, the prevalence of IBD in Mexico is 150,000, and considering an expected prevalence difference and an acceptable error of 5%, the following calculations were made ^9, 10^:

N=[Zα22[p[1−p]]d2]

N = Sample size calculation; Zα / 2 = Z value of the alpha error with 95% confidence, assigning alpha = 0.05; P = expected population prevalence for the event under study (Yamamoto *et al.*, 2015); d = Difference between the expected population prevalence value and the acceptable error. Values: Zα / 2 = 1.96; p = 0.38; d = 0.30.

$$N=\left[\frac{1.96^{2}\left[0.38\left[1-0.38\right]\right]}{0.30^{2}}\right]=384$$

Which means that if you survey 384 people, the actual data you are looking for will be 95% of the time in the ± 5% range relative to the data you see in the survey.

### Data collection

A database was produced with Excel v19.0. The period of data collection was August to September 2020, where the following data were collected from each patient: 1) Type of IBD (UC and CD), 2) Sex of the patient, 3) Health System where they were treated, 4) Are you familiar with the non-invasive stool test to see if the intestine is inflamed? 5) Do you know of any test to determine if the disease is in the active phase? 6) Do you know of any test to determine if the disease is in the remission phase? 7) Do you consider that colonoscopy is necessary for the follow-up of IBD? 8) Do you know the FC test? 9) Have you ever had the FC test? 10) Does your medical unit have the FC test? 11) How accessible is it to perform the FC test in your medical unit? 12) How often does the specialist doctor suggest that you take the FC test? 13) Do you know approximately the cost of the FC test? 14) Do you think the price of the FC test is lower than the endoscopy study?, 15) For the follow-up of the disease would you prefer to perform Colonoscopy or the FC test?

### Statistical analysis

Initially, a univariate analysis was carried out, where absolute and relative frequencies were used for qualitative variables and mean and standard deviation were used for quantitative variables. All tests were performed with Excel v19.0.

### Ethical considerations

This study was based on the guidelines for clinical research established in the Declaration of Helsinki, in the Ministry of Health, and in the “Centro Médico Nacional, 20 de Noviembre, ISSSTE”. The Institutional Biosafety, Ethics and Research Committee, approved this study (number 033.2017). Written informed consent was obtained from each patient. As well as in the Biosafety, Ethics and Institutional Research Committees, patient information was deidentified and data were stored in a confidential registry of Hospital C.M.N. “20 de Noviembre”, ISSSTE.

## Results

### Population characteristics

**Figure 1.**
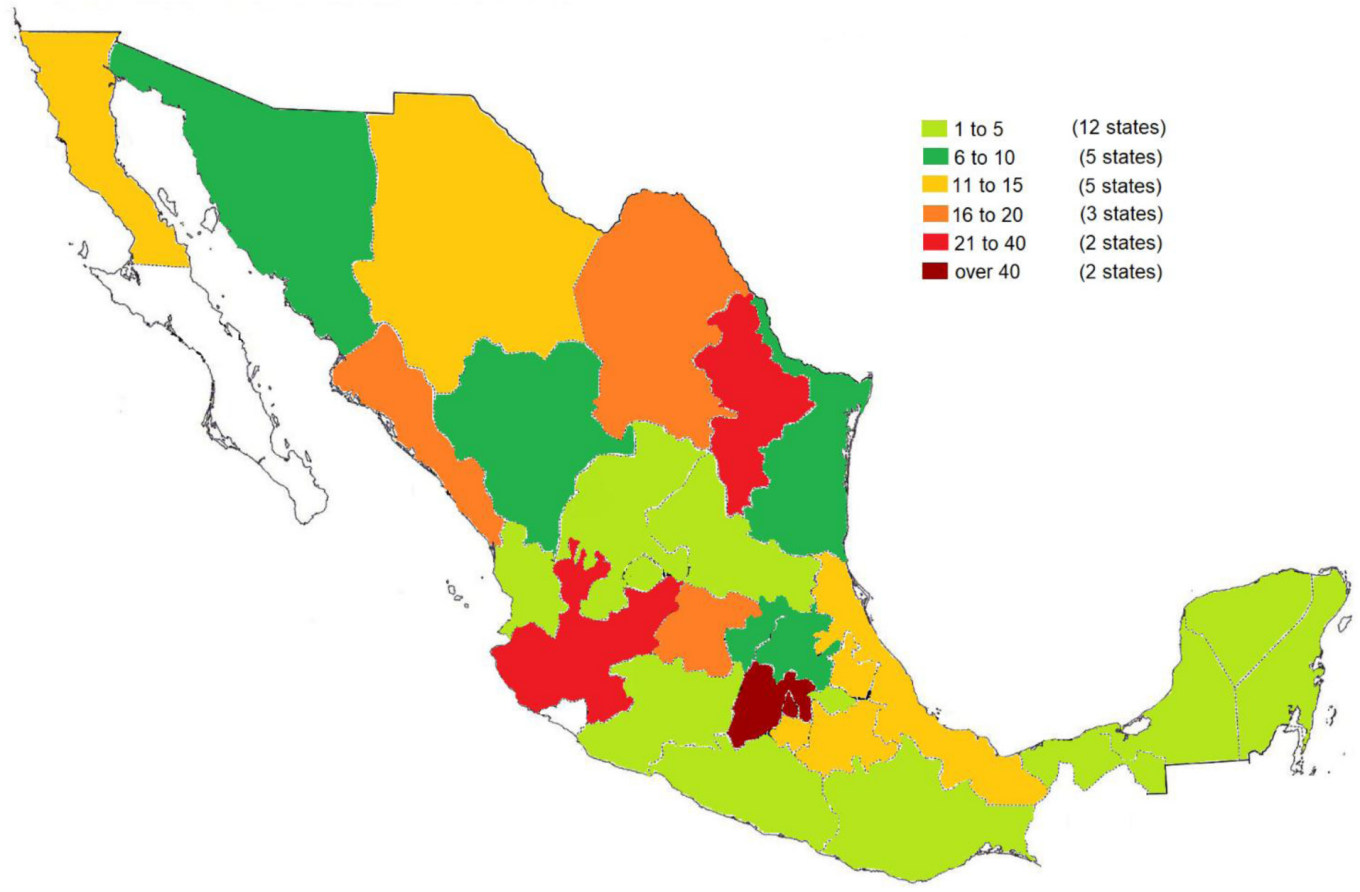
States of the Mexican Republic from which participants took part. The colours represent the number of participants recruited in each state. Mexico City and the State of Mexico (dark red) presented the greatest number of participants (over 40) with IBD. Monterrey and Guadalajara (red) presented 21 to 40 participants with IBD. The states of Coahuila, León and Sinaloa (orange) presented 21 to 40 participants with IBD. Puebla, Morelos, Veracruz, Baja Calicfornia and Chihuahua (yellow) presented 11 to 15 participants with IBD. Pachuca, Querétaro, Ciudad Victoria, Hermosillo and Durango (dark green) presented 6 to 10 participants with IBD. Chilpancingo, Nayarit, Oaxaca, Quintana Roo, San Luis Potosí, Tlaxacala, Yucatán, Aguascalientes, Campeche, Michoacán, Zacatecas and Tabasco (light green) presented 1 to 5 participants with IBD.

In total, 460 patients with a diagnosis of IBD were obtained in 29 states of the Mexican Republic ^11^. The following states and cities of the Mexican Republic were the ones that provided the information: Mexico City, Mexico City (131); State of Mexico, Toluca de Lerdo (68); Monterrey, Nuevo Leon (36); Guadalajara, Jalisco (32); Coahuila, Saltillo (20); Leon, Guanajuato (16); Sinaloa, Culiacán (16); Puebla, Puebla (15); Morelos, Cuernavaca (12); Veracruz, Xalapa (12); Baja California, Mexicali (11); Chihuahua, Chihuahua (11); Pachuca, Hidalgo (9); Querétaro, Querétaro (8); Ciudad Victoria, Tamaulipas (8); Hermosillo, Sonora (7); Durango, Durango (6); Chilpancingo, Guerrero (5); Nayarit, Tepic (5); Oaxaca, Oaxaca (5); Quintana Roo, Chetumal (5); San Luis Potosí, San Luis Potosí (5); Tlaxcala, Tlaxcala (5); Yucatán, Mérida (3); Aguascalientes, Aguascalientes (2); Campeche, Campeche (2); Michoacán, Morelia (2); Zacatecas, Zacatecas (2); Tabasco, Villahermosa (1) ( Figure 1).

Table 1 shows the general characteristics of the study participants. Of the 460 patients who participated in the questionnaire, there was a greater number of patients with a diagnosis of ulcerative colitis (386; 83.9%) than Crohn’s disease (74, 16.0%), and a predominance of women (327, 71%) over men (133, 28.9%), as well as greater medical attention in the public health sector.

**Table 1.** General characteristics of the study population.

	Ulcerative Colitis (UC)	Crohn’s disease (CD)
Number of participants (%)	83.9 % (386)	16.0 % (74)
Sex W/M	273/113	54/20
Age	42.5±11.4	48.6±5.6
Healthcare system Private/Public	165/221	25/49

Data are presented as number (%), and median/±

### Participant knowledge of IBD tests

It was observed that more than 50% of participants were not aware of any non-invasive stool test ( *i.e.* not endoscopy) to test whether the intestine is inflamed. Additionally, more than 50% stated that they did not know of any test to differentiate whether the disease is in the active or remission phase. Moreover, 90% of participants stated that colonoscopy was necessary for the follow-up of IBD ( Table 2).

**Table 2.** Participant knowledge of IBD tests.

	Ulcerative Colitis (UC) n= 386 (83.9 %)	Crohn’s disease (CD) n= 74 (16 %)
Are you familiar with the non-invasive stool test to see if the intestine is inflamed? Yes No	161 (41.7 %) 225 (58.2 %)	32 (43.2 %) 42 (56.7 %)
Do you know of any tests to know if it is in the active phase? Yes No	142 (36.7 %) 244 (63.2 %)	32 (43.2 %) 42 (56.7 %)
Do you know of any test to know if it is in the remission phase? Yes No	120 (31.0 %) 266 (68.9 %)	32 (43.2 %) 42 (56.7 %)
Do you consider that colonoscopy is necessary for the follow-up of IBD? Yes No	352 (91.1 %) 34 (8.8 %)	67 (90.5 %) 7 (9.4 %)

Data are presented as number (%)

### Participant knowledge of the fecal calprotectin test

It was observed that more than 50% of participants had no knowledge of the FC test and have never had it. Furthermore, 64% of participants did not know if their medical unit has the FC test and 83% consider the test hard to access in their medical unit. Additionally, more than 65% of the participants stated that they had never been offered the FC test. Despite this, almost 90% of patients said they would prefer to use the FC test to monitor their condition ( Table 3).

**Table 3.** Participant knowledge of the non-invasive Fecal Calprotectin test.

	Ulcerative Colitis (UC) n= 386 (83.9 %)	Crohn’s disease (CD) n= 74 (16 %)
Are you familiar with the test called Fecal Calprotectin? Yes No	164 (42.4 %) 222 (57.5 %)	31 (41.8 %) 43 (58.1 %)
Have you ever had a Fecal Calprotectin test? Yes No	132 (34.1 %) 254 (65.8 %)	36 (48.6 %) 38 (51.3 %)
How often does the specialist doctor suggests performing the FC test? Every 3–4 months Every 6 months Once a year One time only to prescribe another medicine Never	35 (9.0 %) 39 (10.1 %) 27 (6.9 %) 30 (7.7 %) 255 (66.0 %)	6 (8.1 %) 9 (12.1 %) 8 (10.8 %) 12 (16.2 %) 39 (52.7 %)
Does your medical unit have the FC test? Yes No I don’t know	65 (16.8 %) 74 (19.1 %) 247 (63.9 %)	17 (22.9 %) 19 (25.6 %) 38 (51.3 %)
How accessible is it to take the FC test in your medical unit? Hard Easy	319 (82.6 %) 67 (17.3 %)	57 (77.0 %) 17 (22.9 %)
Do you know the approximate cost of the FC test? Yes No	103 (26.6 %) 283 (73.3 %)	23 (31.0 %) 51 (68.9 %)
Do you think the price of the FC test is less than the endoscopy study? Yes No	274 (70.9 %) 112 (29.01 %)	51 (68.9 %) 23 (31.0 %)
For the follow-up of the disease you prefer to perform: Colonoscopy Fecal Calprotectin	42 (10.8 %) 344 (89.1 %)	9 (12.1 %) 65 (87.8 %)

Data are presented as number (%)

## Discussion

To ensure high quality care for patients with a chronic disease, it is important for the patient to have adequate information on their diagnosis, treatment and follow-up, and for the doctor to inform and discuss different options with the patient. Determining patient knowledge of their disease can help reduce costs in the health sector and at the same time improve the quality of life of patients. Assessing knowledge of fecal calprotectin (CF) in patients with IBD is, therefore, important to improve patient care for this chronic disease. This study is the first to our knowledge to evaluate this in Mexico.

Participants were recruited through the “Fundación Vivir con CU y Crohn SA”, and 460 patients with a diagnosis of IBD were surveyed, of which, 83.9% were diagnosed with UC and 16.0% with CD. The predominance of UC over CD is similar to that reported in other countries in Asia and Latin American countries, such as Colombia ^7, 11^. Although the trend of IBD is similar to that of other countries, the lack of epidemiological data in Mexico remains a challenge to understand the real number of IBD cases. The predominance of women (327, 71%) over men (133, 28.9%), this imbalance may be due to environmental factors such as the use of oral contraceptives, psychosocial stress, dietary factors, among others. On the other hand, it has been described that the use of social networks is more frequent in women than in men, so it can also be attributed that there is a greater number of women ^12– 16^.

In the first category of questioning about the participants’ knowledge of IBD tests, participants mostly reported to not know of any test for the state of the disease. This shows that the majority of participants were uninformed, which can lead to a deterioration in the quality of life ^17^. When living with a chronic disease, it is essential to know about advances in medicine, to know the signs and symptoms, and how to better diagnose and monitor the disease to improve quality of life. The benefits of staying properly informed include awareness of useful tools and tests to diagnose and prevent the advancement of the disease ^11, 18, 19^.

More than 90% of the participants stated that colonoscopy is necessary for the follow-up of IBD. Colonoscopy can be used in the initial differential diagnosis, in the surveillance of carcinoma, and to evaluate abnormalities on imaging tests. However, undergoing a colonoscopy is invasive, expensive, and intolerable for some patients. In other studies, patients have claimed to have anxiety before and during the colonoscopy, given potential risks such as intestinal perforation. Colonoscopy has also been associated with several unfavorable outcomes, including missed diagnoses and avoidable repeat procedures ^20, 21^. In addition, it is difficult to perform an endoscopic evaluation when there is injury to the intestinal mucosa, a frequent problem in IBD ^22– 24^.

In the second part of the questionnaire to identify the degree of knowledge of the CF test, more than 50% of the population in both groups answered that they had no knowledge of the FC test. They also said that the FC test had never been performed on them, and that their specialist doctor had never suggested performing the FC test. This highlights the scarcity of knowledge regarding this test in treating patients with IBD, and that the doctor-patient relationship seems deficient. The doctor must inform the patient of their pathology, the procedures to follow, the treatment possibilities, eventual healing and, in general, must adhere to correct clinical practice to improve the information perceived by patients ^25, 26^.

In addition, it must be clear that the only effective information is that provided by the health professional before the intervention or treatment in question. Adequate information should be provided sufficiently in advance and be honest and easy to understand, so that the patient can make an informed decision ^27^. That is why the dissemination of information to the patient, including the benefits and disadvantages of any potential procedure should be made known ^28^.

The study participants were questioned about the accessibility of the FC test in their medical unit. More than 70% stated that it was difficult to access this test, and in contrast, more than 50% of the population reported not knowing whether this test is available in their medical unit. The Mexican Consensus for the diagnosis and treatment of IBD suggests that the FC test is useful to evaluate the activity of IBD. The test’s response to medical treatment correlates with mucosal scarring or endoscopic remission, and is a good predictor of relapse, so access should be easily available in any medical unit ^29^. Furthermore, expert guidelines suggest that disease activity should be reassessed every 3 to 6 months ^30^, therefore, it would be important for public and private medical units to have the FC test for regular cost-effective testing ^22, 31^.

In questions about the cost of FC, more than 60% of participants stated that they did not know the cost of the test, and more than 60% believed that the price of FC is less than a colonoscopy. On the contrary, a study carried out by Motaganahalli *et al.,* in 2019, suggests that the introduction of the FC test in the routine health care of IBD not only helps the patient, but is potentially cheaper than a colonoscopy ^32^. The use of colonoscopies could be reduced by more than 80% if the FC test is used as the first approach ^33^. In addition, the use of the FC test over a colonoscopy would be more profitable, indirectly, for the patient, since colonoscopies cause loss of productivity and absence from work, require sedation and attendance at a medical care center ^34, 35^.

Finally, the participants were questioned about which test they would prefer to carry out for an IBD follow-up appointment. More than 80% stated that they would prefer the FC test. FC could not only be useful in the differentiation of patients with active IBD and those in remission, but it also correlates well with the degree of inflammatory activity evaluated by clinical indices such as endoscopy and histology, and predicts clinical relapse, as well as the mucosal healing and postoperative recurrence ^36, 37^. Other authors have shown that FC levels are associated with the presence of histological alterations in endoscopic biopsies, being lower in the absence of anatomopathological inflammation ^38– 40^. In different cohorts it has also been reported that the FC test is able to distinguish IBD from Irritable Bowel Syndrome ^41, 42^. Therefore, the use of an alternative non-invasive biomarker such as FC that can accurately differentiate functional or organic diseases, detect inflammation of the intestinal mucosa, and monitor disease activity to avoid invasive and costly testing could be highly useful ^43, 44^. Likewise, this information cannot necessarily be omitted, since clinical guidelines such as that of European Crohn’s and Colitis Organization [ECCO] the European Society of Gastrointestinal, Abdominal Radiology [ESGAR], British Society of Gastroenterology consensus guidelines and Mexican guidelines for the Diagnosis and Treatment of IBD in the Adult Population, recommend the use of the fecal calprotectin test in various clinical settings, including initial diagnosis, relapse diagnosis and response to treatment ^45– 48^.

Assessing patient knowledge of non-invasive tests such as FC in patients with IBD may help to improve patient care as well as a reduction in medical costs during follow-up of the disease. This study suggests that knowledge of such tests can be improved with better communication with the medical personnel. The limitations of the study were that there was a much greater number of participants with ulcerative colitis than Crohn’s disease, and the patients were only evaluated cross-sectionally. On the other hand, there is growing evidence suggesting that Facebook is a useful recruiting tool and therefore its use should be considered when implementing future health research. However, one of the important limitations of this study is Internet access.

In general, patients with chronic diseases should know about the tests that help to diagnose and monitor their disease. For IBD, assessing a biological biomarker, such as FC, is more specific than clinical indices, less expensive, more comfortable than endoscopic monitoring, and reduces the need for endoscopic examinations. Therefore, patients with IBD should be given the correct information on the use of FC which could be a valid alternative to evaluate the response to treatment and reduce the number of colonoscopies performed. To improve knowledge transfer to patients, we suggest improving accessibility to services, creating comprehensive care units, having medical specialists inform their patients about management of the disease, promoting medical research on the disease, and likewise, raising more awareness in society about IBD.

## Conclusion

Using the application of a questionnaire, we evaluated the knowledge of patients with IBD about the diagnostic tests that are required for the diagnosis and follow-up of the disease. There was a lack of awareness of the effective and low-cost Fecal Calprotectin test in the participants. In order for patients to make informed decisions regarding the management of their disease, doctors should provide adequate and timely information about the different options available.

## Data availability

### Underlying data

Harvard Dataverse: Patient knowledge of fecal calprotectin in inflammatory bowel disease (IBD): An observational study in Mexico. https://doi.org/10.7910/DVN/AHPJOF ^11^

This project contains the following underlying data:

- Database Maldonado et al.tab. (Excel spreadsheet of questionnaire responses)

### Extended data

Harvard Dataverse: Patient knowledge of fecal calprotectin in inflammatory bowel disease (IBD): An observational study in Mexico. https://doi.org/10.7910/DVN/NOOUQF ^8^

This project contains the following extended data:

- Questionnaire in English
- Questionnaire in Spanish

Data are available under the terms of the Creative Commons Attribution 4.0 International license (CC-BY 4.0).

## Consent

Written informed consent for publication of the participants’ details was obtained from participants.
