# Supplementary material for: Patient knowledge of fecal calprotectin in inflammatory bowel disease (IBD): An observational study in Mexico
Source: F1000Res. 2021 Feb 5;9:1496. Originally published 2020 Dec 21. [Version 2] doi: 10.12688/f1000research.27629.2 (PMC9418753; doi:10.12688/f1000research.27629.2)
Supplement: Peer review history

# Peer review: Reviewer response for version 2

**DOI:** 10.5256/f1000research.54322.r144061

**Published:** 2022-08-26

**Authors:** Rupert W. Leong

**Affiliations:** 1 Concord Clinical School, University of Sydney, Sydney, NSW, Australia

The article is relevant to local practice in the management of IBD patients. It is not generalisable based on the sample size and questionnaire design, but the main point of the study is to reflect on opportunities for treatment improvement in Mexico. Although there was a high proportion of UC versus CD it most likely reflects the local epidemiology, as previously documented in a review ^1^.

 The lack of awareness of non-invasive testing is obviously multi-factorial. Awareness might not drive increased uptake due to the cost of testing faecal calprotectin, acceptability of testing, tissue processing, accuracy of results, timely reporting and use of the results by clinicians. All these are important factors before the test is widely endorsed and the effectiveness of real-world testing needs to be conducted locally. There is no doubt internationally that faecal calprotectin is an important target of treatment in IBD and in countries where colonoscopy is not widely-available the importance of faecal calprotectin is further accentuated.

Is the work clearly and accurately presented and does it cite the current literature?

Yes

If applicable, is the statistical analysis and its interpretation appropriate?

No

Are all the source data underlying the results available to ensure full reproducibility?

Partly

Is the study design appropriate and is the work technically sound?

Partly

Are the conclusions drawn adequately supported by the results?

Yes

Are sufficient details of methods and analysis provided to allow replication by others?

Yes

Reviewer Expertise:

NA

I confirm that I have read this submission and believe that I have an appropriate level of expertise to confirm that it is of an acceptable scientific standard.

## References

[1] : Epidemiology of inflammatory bowel disease in South America: A systematic review. *World J Gastroenterol* .2019;25(47) : 10.3748/wjg.v25.i47.6866 6866-6875 10.3748/wjg.v25.i47.6866 31885427PMC6931006

# Peer review: Reviewer response for version 2

**DOI:** 10.5256/f1000research.54322.r79014

**Published:** 2021-02-12

**Authors:** Javier P Gisbert

**Affiliations:** 1 Departamento de Medicina, Universidad Autónoma de Madrid, Madrid, Spain

I think that the article is now ready to be indexed.

Is the work clearly and accurately presented and does it cite the current literature?

Yes

If applicable, is the statistical analysis and its interpretation appropriate?

Partly

Are all the source data underlying the results available to ensure full reproducibility?

Yes

Is the study design appropriate and is the work technically sound?

Yes

Are the conclusions drawn adequately supported by the results?

Partly

Are sufficient details of methods and analysis provided to allow replication by others?

Partly

Reviewer Expertise:

NA

I confirm that I have read this submission and believe that I have an appropriate level of expertise to confirm that it is of an acceptable scientific standard.

# Peer review: Reviewer response for version 1

**DOI:** 10.5256/f1000research.30540.r76438

**Published:** 2021-01-06

**Authors:** Javier P Gisbert

**Affiliations:** 1 Departamento de Medicina, Universidad Autónoma de Madrid, Madrid, Spain

**TITLE**

 OK.

**ABSTRACT**

 OK.

**INTRODUCTION**

 OK.

**METHODS**

 It seems that the questionnaire had been previously used in another study (as a reference is provided for it). Was it previously validated? Please clarify this.

 As the questionnaire was posted on Facebook site, there may be a bias for including only patients with internet access (which could be associated, for example, with younger age, higher educational status, etc.). This potential limitation should be discussed in the Discussion section.

 In this respect, inclusion criteria were Mexican adults, aged 18 to 45 years old. Please justify this. This could induce another bias, as older patients could have lower internet access. Therefore, the results of the present study could have been biased to more favorable findings. This potential limitation should also be discussed in the Discussion section.

 It is stated that the sample size was calculated using the central limit theorem with a margin of error of 5% and a confidence interval of 95%. However, more details should be provided, and the calculated sample size should be given.

 Statistical analysis: It is stated that “Initially, a univariate analysis was carried out”. This seems to imply that a multivariate analysis was also performed, but I cannot see any information regarding it.

**RESULTS**

 Of the 460 patients who participated in the questionnaire, there was a much greater number of patients with a diagnosis of ulcerative colitis (386; 83.9%) than Crohn’s disease (74, 16.0%). Even though the authors point out that “The predominance of UC over CD is similar to that reported in other countries in Asia and Latin American countries, such as Colombia”, and they comment in the Discussion section that “The limitations of the study were that there was a much greater number of participants with ulcerative colitis than Crohn’s disease”, these explanations seem to be insufficient. Please try to explain better this possible bias.

 The same is valid for the predominance of women (327, 71%) over men (133, 28.9%), please try to explain this misbalance.

**DISCUSSION**

 It is repeatedly stated that: “The majority of participants were uninformed, which can lead to a deterioration in the quality of life”. And that “They also said that the FC test had never been performed on them and that their specialist doctor had never suggested performing the FC test. This highlights the scarcity of knowledge regarding this test in treating patients with IBD, and that the doctor-patient relationship seems deficient”. However, it should also be clarified that the lack of knowledge of the patient does not necessarily imply that the doctor has not informed the patient correctly, since it is possible that the determination of FC is simply not indicated in them. In other words, it is clear that FC is not always indicated in all patients, and for those patients, this information could be correctly omitted. Please clarify this in the Discussion section.

**REFERENCES**

 OK.

**TABLES**

 OK.

**FIGURES**

 OK.

Is the work clearly and accurately presented and does it cite the current literature?

Yes

If applicable, is the statistical analysis and its interpretation appropriate?

Partly

Are all the source data underlying the results available to ensure full reproducibility?

Yes

Is the study design appropriate and is the work technically sound?

Yes

Are the conclusions drawn adequately supported by the results?

Partly

Are sufficient details of methods and analysis provided to allow replication by others?

Partly

Reviewer Expertise:

Inflammatory bowel disease

I confirm that I have read this submission and believe that I have an appropriate level of expertise to confirm that it is of an acceptable scientific standard, however I have significant reservations, as outlined above.

# Response

**Published:** 2021-01-20

**Authors:** Brenda Maldonado

**Affiliations:** Escuela Nacional de Ciencias Biológicas, Mexico

Dear Reviewer 1:

 METHODS

 Reviewer 1: It seems that the questionnaire had been previously used in another study (as a reference is provided for it). Was it previously validated? Please clarify this.

 Answer: *Thanks for the comment. The questionnaire was developed by “Fundación Vivir con Crohn y CUCI A.C”, gastroenterologists and health advocates of Hospital C.M.N. "20 de Noviembre", ISSSTE, in 2020. It is an original questionnaire, without prior use. Two gastroenterologists in the group validated the 15 questions. A pilot test of the questionnaire was carried out with 5% of the population (23 people with IBD who attended the hospital), to ensure the validity of the questionnaire.*

 Reviewer 1: As the questionnaire was posted on Facebook site, there may be a bias for including only patients with internet access (which could be associated, for example, with younger age, higher educational status, etc.). This potential limitation should be discussed in the Discussion section.

 Answer: *Thank you for the suggestion. It will be added as a limitation in the discussion.*

*……Although there is growing evidence to suggest that Facebook is a useful recruiting tool and therefore its use should be considered when implementing future health research. However, one of the important limitations of this study is Internet access….*

*Whitaker, C., Stevelink, S., & Fear, N. (2017). The Use of Facebook in Recruiting Participants for Health Research Purposes: A Systematic Review. Journal of medical Internet research, 19(8), e290. https://doi.org/10.2196/jmir.7071*

 Reviewer 1: In this respect, inclusion criteria were Mexican adults, aged 18 to 45 years old. Please justify this. This could induce another bias, as older patients could have lower internet access. Therefore, the results of the present study could have been biased to more favorable findings. This potential limitation should also be discussed in the Discussion section.

 Answer: *Thanks for the suggestion. The inclusion criterion based on the age of 18 to 45 years of age, was chosen because in previous studies it is presented primarily to people of productive age (Sairenji, T., Collins, K. L., & Evans, D. V. (2017). An Update on Inflammatory Bowel Disease. Primary care, 44(4), 673–692. https://doi.org/10.1016/j.pop.2017.07.010. Kamp, K., Dudley-Brown, S., Heitkemper, M., Wyatt, G., & Given, B. (2020). Symptoms among emerging adults with inflammatory bowel disease: a descriptive study. Research in nursing & health, 43(1), 48–55. https://doi.org/10.1002/nur.21985)*

 Reviewer 1: It is stated that the sample size was calculated using the central limit theorem with a margin of error of 5% and a confidence interval of 95%. However, more details should be provided, and the calculated sample size should be given.

 Answer: *According to a previous study by Yamamoto et al., 2015, the prevalence of IBD in Mexico is 150,000, and considering an expected prevalence difference and an acceptable error of 5%, the following calculations were made:*

*N=Zα22p1-pd2*

*N = Sample size calculation; Zα / 2 = Z value of the alpha error with 95% confidence, assigning alpha = 0.05; P = expected population prevalence for the event under study (Yamamoto et al., 2015); d = Difference between the expected population prevalence value and the acceptable error. Values: Zα / 2 = 1.96; p = 0.38; d = 0.30.*

*N= 1.9620.381-0.380.302= 384*

*This means that if you survey 384 people, the actual data you are looking for will be 95% of the time in the ± 5% range relative to the data you see in the survey.*

 Reviewer 1: Statistical analysis: It is stated that “Initially, a univariate analysis was carried out”. This seems to imply that a multivariate analysis was also performed, but I cannot see any information regarding it.

 Answer: *Thank you for your comment. Only univariate analysis was performed*

 RESULTS

 Of the 460 patients who participated in the questionnaire, there was a much greater number of patients with a diagnosis of ulcerative colitis (386; 83.9%) than Crohn’s disease (74, 16.0%). Even though the authors point out that “The predominance of UC over CD is similar to that reported in other countries in Asia and Latin American countries, such as Colombia”, and they comment in the Discussion section that “The limitations of the study were that there was a much greater number of participants with ulcerative colitis than Crohn’s disease”, these explanations seem to be insufficient. Please try to explain better this possible bias.

 Answer: *Thanks for the suggestion. We add the explanation*

 “The limitations of the study were that there was a much greater number of participants with ulcerative colitis than Crohn’s disease”. Although the trend of IBD is similar to other countries, the lack of epidemiological data in Mexico is still a challenge to understand the true number of IBD cases.

 Yamamoto-Furusho, J. K., Bosques-Padilla, F. J., Charúa-Guindic, L., Cortés-Espinosa, T., Miranda-Cordero, R. M., Saez, A., & Ledesma-Osorio, Y. (2020). Inflammatory bowel disease in Mexico: Epidemiology, burden of disease, and treatment trends. Epidemiología, carga de la enfermedad y tendencias de tratamiento de la enfermedad inflamatoria intestinal en México. *Revista de gastroenterologia de Mexico*, *85*(3), 246–256. https://doi.org/10.1016/j.rgmx.2019.07.008

 The same is valid for the predominance of women (327, 71%) over men (133, 28.9%), please try to explain this misbalance.

 Answer: *Thanks for the suggestion. We add the explanation*

 The predominance of women (327, 71%) over men (133, 28.9%), this imbalance may be due to environmental factors such as the use of oral contraceptives, psychosocial stress, dietary factors, among others. On the other hand, it has been described that the use of social networks is more frequent in women than in men, so it can also be attributed that there is a greater number of women.

 Grosberg, D., Grinvald, H., Reuveni, H., & Magnezi, R. (2016). Frequent Surfing on Social Health Networks is Associated With Increased Knowledge and Patient Health Activation. *Journal of medical Internet research*, *18*(8), e212. https://doi.org/10.2196/jmir.5832

 Algethmi, W., Baumann, C., Alnajjar, W., Sroji, A., Alsahafi, M., Jawa, H., Qari, Y., Peyrin-Biroulet, L., Saadah, O. I., & Mosli, M. (2020). Environmental exposures and the risk of inflammatory bowel disease: a case-control study from Saudi Arabia. *European journal of gastroenterology & hepatology*, *32*(3), 358–364. https://doi.org/10.1097/MEG.0000000000001619

 Wang, X., Fan, X., Deng, H., Zhang, X., Zhang, K., Xu, J., Li, N., Han, Q., & Liu, Z. (2019). Use of oral contraceptives and risk of ulcerative colitis - A systematic review and meta-analysis. *Pharmacological research*, *139*, 367–374. https://doi.org/10.1016/j.phrs.2018.11.036

 Ho, S. M., Lewis, J. D., Mayer, E. A., Plevy, S. E., Chuang, E., Rappaport, S. M., Croitoru, K., Korzenik, J. R., Krischer, J., Hyams, J. S., Judson, R., Kellis, M., Jerrett, M., Miller, G. W., Grant, M. L., Shtraizent, N., Honig, G., Hurtado-Lorenzo, A., & Wu, G. D. (2019). Challenges in IBD Research: Environmental Triggers. *Inflammatory bowel diseases*, *25*(Suppl 2), S13–S23. https://doi.org/10.1093/ibd/izz076

 DISCUSSION

 It is repeatedly stated that: “The majority of participants were uninformed, which can lead to a deterioration in the quality of life”. And that “They also said that the FC test had never been performed on them and that their specialist doctor had never suggested performing the FC test. This highlights the scarcity of knowledge regarding this test in treating patients with IBD, and that the doctor-patient relationship seems deficient”. However, it should also be clarified that the lack of knowledge of the patient does not necessarily imply that the doctor has not informed the patient correctly, since it is possible that the determination of FC is simply not indicated in them. In other words, it is clear that FC is not always indicated in all patients, and for those patients, this information could be correctly omitted. Please clarify this in the Discussion section.

 Answer: *Thanks for the comment. We add the explanation.*

 This information cannot necessarily be omitted, since clinical guidelines such as that of European Crohn’s and Colitis Organization [ECCO] the European Society of Gastrointestinal, Abdominal Radiology [ESGAR], British Society of Gastroenterology consensus guidelines and Mexican guidelines for the Diagnosis and Treatment of IBD in the Adult Population, recommend the use of the fecal calprotectin test in various clinical settings, including initial diagnosis, relapse diagnosis and response to treatment.

 Maaser, C., Sturm, A., Vavricka, S. R., Kucharzik, T., Fiorino, G., Annese, V., Calabrese, E., Baumgart, D. C., Bettenworth, D., Borralho Nunes, P., Burisch, J., Castiglione, F., Eliakim, R., Ellul, P., González-Lama, Y., Gordon, H., Halligan, S., Katsanos, K., Kopylov, U., Kotze, P. G., … European Crohn’s and Colitis Organisation [ECCO] and the European Society of Gastrointestinal and Abdominal Radiology [ESGAR] (2019). ECCO-ESGAR Guideline for Diagnostic Assessment in IBD Part 1: Initial diagnosis, monitoring of known IBD, detection of complications. *Journal of Crohn's & colitis*, *13*(2), 144–164. https://doi.org/10.1093/ecco-jcc/jjy113

 Cannatelli, R., Bazarova, A., Zardo, D., Nardone, O. M., Shivaji, U., Smith, S., Gkoutos, G., Ricci, C., Gui, X. S., Ghosh, S., & Iacucci, M. (2020). Fecal Calprotectin Thresholds to Predict Endoscopic Remission Using Advanced Optical Enhancement Techniques and Histological Remission in IBD Patients. *Inflammatory bowel diseases*, izaa163. Advance online publication. https://doi.org/10.1093/ibd/izaa163

 Krzystek-Korpacka, M., Kempiński, R., Bromke, M., & Neubauer, K. (2020). Biochemical Biomarkers of Mucosal Healing for Inflammatory Bowel Disease in Adults. *Diagnostics (Basel, Switzerland)*, *10*(6), 367. https://doi.org/10.3390/diagnostics10060367

 Ricciuto, A., & Griffiths, A. M. (2019). Clinical value of fecal calprotectin. *Critical reviews in clinical laboratory sciences*, *56*(5), 307–320. https://doi.org/10.1080/10408363.2019.1619159

 We greatly appreciate every suggestion.
